# Rotating night shifts too quickly may cause anxiety and decreased attentional performance, and impact prolactin levels during the subsequent day: a case control study

**DOI:** 10.1186/s12888-014-0218-7

**Published:** 2014-08-05

**Authors:** Yu-San Chang, Hsiang-Lan Chen, Yu-Hsuan Wu, Chung-Yao Hsu, Ching-Kuan Liu, Chin Hsu

**Affiliations:** Graduate Institute of Medicine, College of Medicine, Kaohsiung Medical University, No. 100, Tzyou 1st Road, Kaohsiung, 807 Taiwan; Faculty of Nursing Department, Meiho University, No. 23, Pingguang Road, Neipu, Pingtung, Taiwan; Kaohsiung Municipal Kai-Syuan Psychiatric Hospital, No. 130, Kai-Syuan, 2nd Road, Ling-Ya District, Kaohsiung, 802 Taiwan; Department of Neurology, Kaohsiung Medical University Hospital, No. 100, Tzyou 1st Road, Kaohsiung, 807 Taiwan; Kaohsiung Medical University, No. 100, Tzyou 1st Road, Kaohsiung, 807 Taiwan; Department of Physiology, Kaohsiung Medical University, No. 100, Tzyou 1st Road, Kaohsiung, 807 Taiwan

**Keywords:** Anxiety, Cognitive function, Nurse, Night shift, Sleep-related hormone, Circadian, Shift work

## Abstract

**Background:**

We investigated circadian changes and effects on mood, sleep-related hormones and cognitive performance when nurses worked consecutive night shifts in a rapidly rotating shift system. Daytime cognitive function, sleep propensity and sleep-related hormones (growth hormone, cortisol, prolactin, thyrotropin) were compared after participants worked two and four consecutive night shifts.

**Methods:**

Twenty-three off-duty nurses, 20 nurses working two consecutive night shifts and 16 nurses working four consecutive night shifts were enrolled. All participants completed the Maintenance of Wakefulness Test, State-Trait Anxiety Inventory, Stanford Sleepiness Scale, visual attention tasks (VAT), Wisconsin Card Sorting Test, and modified Multiple Sleep Latency Test. Hormone levels were also measured four times throughout the day, at 2-h intervals.

**Results:**

During the day, the participants in the night shift groups were less able to maintain wakefulness, had poor performance on VAT, and higher thyrotropin levels than did those in the off-duty group. Participants who worked two night shifts were better able to maintain wakefulness, had higher anxiety scale scores, poorer initial performance and lack of learning effect on VAT, and higher prolactin levels compared with those who worked four night shifts. There were no differences in cortisol levels between the two- and four- shift groups.

**Conclusions:**

Rotating night shifts too quickly may cause anxiety and decreased attentional performance, and may impact daytime prolactin levels after night shifts. It is possible that the two-shift group had a higher cortisol level than did the four-shift group, which would be consistent with the group’s higher state anxiety scores. The negative findings may be due to the small sample size. Further studies on the effects of consecutive night shifts on mood and cortisol levels during the daytime after sleep restriction would be valuable.

## Background

Shift work schedules in the medical field vary on several dimensions, including length of work (e.g., 8-h versus 12-h shifts), fixed versus rotating scheduling, and duration of rotation. A slower rotation schedule is one that permits workers to adjust their circadian rhythm gradually over a period of 2 to 4 weeks [1]. In faster rotations (e.g., shifting every 3 to 5 days), research [1] shows that workers will maintain constant circadian rhythms in coordination with the environment. An 8-h shift system with faster rotation is common in the medical field in Taiwan. At our hospital, the night shift work schedule of most nursing staff consists of two to four consecutive night shifts followed by at least 1 day off. During the off day, nurses are expected to adjust their circadian rhythms in preparation for the next daytime shift.

Sleep deprivation research [2] has shown diverse impacts on mood and cognitive performance, and many studies [3–6] have investigated the influence of night shifts on the performance of work at night. However, little is known about the impact of consecutive night shifts on subsequent daytime performance. In addition, sleep deprivation has also been reported to affect sleep-related hormones. Leproult et al. [7] reported that sleep loss appears to delay the normal return to evening quiescence of the corticotropic axis, resulting in increased cortisol levels the following evening compared with the previous evening. Sleep deprivation has also been reported to increase thyrotropin (TSH) to about double the usual level [8]. However, little is known about the impact on sleep-related hormones during the daytime after working different lengths of consecutive night shifts. Research on rapidly rotating night shift systems can provide valuable information regarding circadian changes and effects on mood and cognitive performance.

In this study, therefore, we compared changes in cognitive function, state anxiety, and objectively measured sleep propensity in the daytime after two consecutive night shifts and after four consecutive night shifts. We also measured daytime levels of sleep-related hormones (growth hormone, GH; cortisol; prolactin, PRL; and TSH).

## Methods

### Participants and procedures

The 59 participants included 23 off-duty (OD) female nurses (mean age 26.1 ± 1.9 years; mean years of education 15.3 ± 1.0), 20 female nurses working two consecutive night shifts (2NS; mean age 26.0 ± 2.0 years, mean years of education 14.7 ± 1.0) and 16 female nurses working four consecutive night shifts (4NS; mean age 27.1 ± 2.0 years; mean years of education 15.4 ± 1.0). All participants worked in the acute ward of Kaohsiung Municipal Kai-Syuan Psychiatric Hospital in southern Taiwan. We excluded those who reported any of the following characteristics on a screening questionnaire: current use of hypnotics, regular coffee-drinking, psychiatric illness, major systemic disease or sleep disorder. Most of the work schedules of our nurses consist of repetitive blocks of two consecutive day shifts (8 a.m. to 4 p.m. or 8 a.m. to 5:30 p.m.), two evening shifts (4 p.m. to 12 a.m.), two night shifts (12 a.m. to 8 a.m.), and then at least 1 day off. Occasionally, nurses work night shifts for 3 to 4 days consecutively because of staffing demands. To prevent adaptation to night shifts, all of the nurses had worked either day shifts or been free of duty for at least 3 days before entering the study. Those in the night shift groups were asked to sleep prophylactically from 7 p.m. to 11 p.m. while working nights. Demographic data including age, years of education, and mean self-reported total sleep time (TST, including daytime sleep and prophylactic sleep) for the night shift groups and the sleep time during the night before the study began for the OD group were recorded. This study was approved by the Ethics Committee of Kaohsiung Municipal Kai-Suan Psychiatric Hospital (KSPH-2011-15) and all participants gave written consent after being fully informed of the nature and procedures of the study.

**Figure 1:**
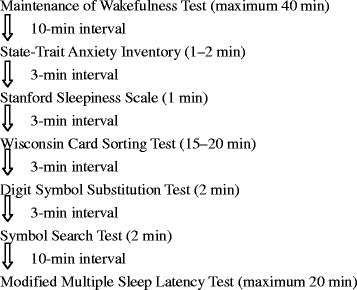
**Schedule for administering measurements.** All participants completed a set of measures every 2 h for a total of four times.

All participants arrived at the sleep laboratory at about 9:00 a.m. after their two or four consecutive night shifts. On this off-duty day, they spent about 8 h in the laboratory completing a set of measures every 2 h for a total of four times, starting at 9:20 a.m. The measures included the Maintenance of Wakefulness Test (MWT), State-Trait Anxiety Inventory (STAI) [9], Stanford Sleepiness Scale (SSS) [10], Wisconsin Card Sorting Test (WCST) [11], Digit Symbol Substitution Test (DSST), Symbol Search Test (SST) [12], and the modified Multiple Sleep Latency Test (modified MSLT) (Figure 1). Bedside blood samples were collected at the end of the MSLT and were tested for sleep-related hormones. The variation in the time of blood sample collection was within 20 min for each participant and each collection, and occurred at 11:00 a.m.–11:20 a.m., 1:00 p.m.–1:20 p.m., 3:00 p.m.–3:20 p.m., and 5:00 p.m.–5:20 p.m. All participants were required to remain awake during the test day, and all of the tests were given individually in the same experimental setting.

### Measurements

The STAI is a self-reported measure of both state and trait anxiety that comprises 20 items, all rated on a four-point scale, with a higher score indicating greater anxiety. The STAI has demonstrated good validity and internal consistency (Cronbach’s α > 0.85 and test-retest reliability ≥ 0.75) [13]. The SSS is a seven-point self-rating scale used to quantify progressive steps in sleepiness, from 1 (alert) to 7 (no longer fighting sleep).

The computerized WCST, which is loosely considered a measure of frontal lobe ability, consists of four stimulus cards and 128 response cards. Cards differ in the color, form and number of shapes they contain. The examinee is asked to match each response card from the deck with whichever of the four stimulus cards seems most appropriate. The dependent variables included number of perseverative errors, total errors, and categories, as well as percentage of conceptual-level responses and failure to maintain set. Both the DSST and SST of visual attention tasks (VAT) are subsets of the Wechsler Adult Intelligence Scale [12] involving cognitive, perceptual and motor abilities. For the DSST, examinees enter appropriate symbols into empty squares beneath digits. In the SST, they respond to one of two target symbols from four selective symbols. The raw scores of the DSST and SST were determined by the number of items correctly completed in 120 seconds, and the raw scores were then converted to a scale score according to age. The information processing index (IPI) was obtained after transforming the sum of the scale scores of the SST and DSST.

The modified MSLT was performed by partial-montage polysomnography consisting of electroencephalography (at F3/A2, F4/A1, C4/A1, C3/A2, O2/A1, O1/A2), electrooculo-graphy, and submental electromyography. We scored sleep records visually according to the American Academy of Sleep Medicine criteria [14]. The participants lay in a quiet, dark bedroom and attempted to fall asleep. They were awoken after 3 epochs of stage one, or immediately when they entered any other sleep stage. The sleep latency was taken as the first epoch of any stage of sleep. If sleep onset did not occur, a latency of 20 min (the end of the test period) was used for data analysis. The MWT was administered in a similar method as the MSLT. The major differences were that in the MWT, the participants were instructed to remain awake, and the termination criterion was 40 min.

Blood samples were collected by venipuncture (5 ml) in vacuum tubes for sleep-related hormone assays. Serum was separated immediately after blood collection, and the samples were stored at −20°C until analysis. Quantitative determination of cortisol, PRL, GH, and TSH levels in the serum was achieved with a paramagnetic particle, chemiluminescent immunometric assay using a Beckman Access system (Beckman Coulter, Inc., Fullerton, CA, USA), Siemens DPC Immulite 2000 analyzer (Siemens Healthcare Diagnostic Products Ltd. Llanberis, Gwynedd, UK), Siemens DPC Immulite 2000 analyzer (Siemens Healthcare Diagnostic Products Ltd.), and Abbott I2000 (Abbott Ireland Diagnostics Division, Longford, Ireland), respectively. The lower limits of detection were 0.4 μg/dl, 0.16 ng/ml, 0.01 ng/ml, and 0.0025 mIU/L, respectively. The intra-assay coefficient of variation averaged 5% for each item.

### Statistical analysis

One-way analysis of variance (ANOVA) was used to compare continuous variables among the three groups. Repeated measures ANOVA was performed, with groups as between-subject factors and time series data as within-subject factors. A *p*-value less than 0.05 was considered statistically significant.

## Results

There were no differences in age (F_(2, 56)_ = 1.77, *p* = 0.179), years of education (F_(2, 56)_ = 2.58, *p* = 0.085), trait anxiety scores (43.9 ± 8.1 vs. 43.0 ± 7.6 vs. 43.8 ± 8.0; F_(2, 56)_ = 0.08, *p* = 0.927), mean TST (6.9 ± 1.1 h vs. 6.7 ± 1.0 h vs. 6. 8 ± 1.2 h; F_(2, 56)_ = 0.18, *p* = 0.833), mean sleep latency (MSL) of the MSLT (*p* = 0.314), SSS scores (*p* = 0.451), GH (*p* = 0.697) or cortisol (*p* = 0.884) levels among the three groups (Tables 1, 2 and 3). There were no significant group differences in any parameters of the WCST. However, significant differences did emerge in the MSL of the MWT (*p* < 0.001), in state anxiety levels (*p* = 0.009), IPI scores (*p* = 0.002), DSST scores (*p* = 0.013), SST scores (*p* = 0.003) and levels of PRL (*p =* 0.003) and TSH (*p* = 0.003) (Tables 1, 2 and 3).

**Table 1:** **Comparison of time series data among the OD (off-duty), 2NS (2 night shifts) and 4NS (4 night shifts) groups**

**Variables (mean ± SD)**	**Group (** ***n*** **= 23, 20, 16)**	**Time 1**	**Time 2**	**Time 3**	**Time 4**	**F(2,56)**	***P-*** **value**
Sleep latency of MWT (min)	Off-duty	24.3 ± 7.6	26.1 ± 7.0	23.4 ± 9.4	22.0 ± 8.3	11.03	<0.001^a^
	Two night shifts	17.0 ± 13.1	22.1 ± 15.1	18.8 ± 12.9	16.3 ± 14.8		
	Four night shifts	8.2 ± 6.9	11.0 ± 12.2	13.1 ± 11.6	10.1 ± 13.7		
Sleep latency of MSLT (min)	Off-duty	13.2 ± 6.7	10.4 ± 6.4	8.6 ± 6.3	8.3 ± 5.9	1.18	0.314
	Two night shifts	10.0 ± 6.3	8.3 ± 6.1	8.0 ± 6.3	7.4 ± 5.2		
	Four night shifts	7.5 ± 5.2	6.0 ± 4.6	8.5 ± 7.2	8.6 ± 7.4		
State Anxiety Scale	Off-duty	39.7 ± 7.1	38.4 ± 6.8	36.5 ± 8.1	35.4 ± 8.5	5.18	0.009^b^
	Two night shifts	42.5 ± 8.0	43.6 ± 7.4	43.4 ± 7.3	42.2 ± 7.7		
	Four night shifts	38.6 ± 7.0	36.8 ± 7.8	35.1 ± 7.5	33.9 ± 7.0		
Stanford Sleepiness Scale	Off-duty	3.2 ± 0.2	3.5 ± 0.2	3.8 ± 0.2	4.1 ± 0.2	0.81	0.451
	Two night shifts	3.4 ± 1.0	3.7 ± 1.1	4.2 ± 1.3	4.3 ± 1.3		
	Four night shifts	3.4 ± 1.0	3.4 ± 1.0	3.4 ± 1.0	3.9 ± 1.3		
Wisconsin Card Sorting Test							
Number of perseverative errors	Off-duty	11.6 ± 4.3	9.7 ± 2.9	10.7 ± 2.7	9.8 ± 2.5	0.98	0.383
	Two night shifts	12.1 ± 6.9	11.5 ± 6.4	12.1 ± 7.3	12.8 ± 11.0		
	Four night shifts	9.9 ± 2.6	9.9 ± 1.9	10.25 ± 4.1	10.6 ± 3.1		
Number of total errors	Off-duty	23.5 ± 7.2	20.7 ± 5.7	18.3 ± 4.9	17.2 ± 5.1	1.44	0.246
	Two night shifts	24.7 ± 14.0	22.4 ± 12.0	23.0 ± 12.5	24.0 ± 13.7		
	Four night shifts	21.2 ± 5.2	19.6 ± 5.3	19.6 ± 5.6	18.2 ± 4.5		

Abbreviations: *MWT* Maintenance of Wakefulness Test, *MSLT* Multiple Sleep Latency Test, *SD* standard deviation.

Scheffé’s post-hoc test: ^a^OD group > 2NS group, OD group > 4NS group, 2NS group > 4ND group; ^b^OD group < 2NS group, 4NS group < 2NS group.

**Table 2:** **Comparison of time series data among the OD (off-duty), 2NS (two night shifts) and 4NS (four night shifts) groups**

**Variables (mean ± SD)**	**Group (** ***n*** **= 23, 20, 16)**	**Time 1**	**Time 2**	**Time 3**	**Time 4**	**F(2,56)**	***P-*** **value**
Wisconsin Card Sorting Test							
Number of categories	Off-duty	7.6 ± 1.9	8.0 ± 1.5	8.5 ± 1.3	8.8 ± 1.2	0.08	0.926
	Two night shifts	7.9 ± 2.3	8.4 ± 2.2	8.2 ± 2.3	8.1 ± 2.2		
	Four night shifts	8.4 ± 1.4	8.3 ± 1.2	8.3 ± 1.3	8.3 ± 1.1		
% of conceptual level responses	Off-duty	73.7 ± 14.5	79.4 ± 8.9	81.7 ± 8.2	84.7 ± 5.9	0.81	0.449
	Two night shifts	76.7 ± 15.7	79.4 ± 13.1	78.8 ± 15.0	77.6 ± 14.8		
	Four night shifts	81.2 ± 7.1	82.0 ± 6.7	81.6 ± 6.0	83.8 ± 3.1		
Failure to maintain set	Off-duty	1.2 ± 0.9	1.8 ± 1.4	1.4 ± 1.3	1.4 ± 1.4	2.47	0.094
	Two night shifts	1.2 ± 0.8	1.6 ± 1.2	1.2 ± 1.1	1.3 ± 1.6		
	Four night shifts	1.8 ± 1.3	1.8 ± 1.3	1.7 ± 1.3	2.3 ± 1.3		
Digit Symbol Substitution Test							
Scoring scale	Off-duty	14.7 ± 1.6	15.2 ± 1.9	15.6 ± 1.9	16.5 ± 2.1	4.72	0.013^a^
	Two night shifts	13.3 ± 2.8	14.0 ± 2.7	14.4 ± 2.8	14.4 ± 2.9		
	Four night shifts	12.1 ± 3.5	13.4 ± 2.9	14.0 ± 2.8	14.2 ± 2.0		
Symbol Searching Test							
Scoring scale	Off-duty	14.4 ± 1.1	15.5 ± 2.6	15.6 ± 2.0	16.7 ± 1.8	6.28	0.003^a^
	Two night shifts	13.3 ± 3.2	13.8 ± 2.4	14.4 ± 3.1	14.9 ± 2.8		
	Four night shifts	11.7 ± 4.1	12.8 ± 3.6	12.7 ± 3.5	14.1 ± 2.8		
Information process index	Off-duty	126.0 ± 7.7	130.3 ± 10.7	131.5 ± 9.2	137.7 ± 9.0	7.21	0.002^a^
	Two night shifts	118.0 ± 14.6	121.6 ± 13.2	124.6 ± 14.7	126.0 ± 13.7		
	Four night shifts	110.9 ± 18.1	117.1 ± 17.2	116.1 ± 18.8	123.9 ± 12.6		

Scheffé’s s post-hoc test: ^a^OD group > 2NS group, OD group > 4NS group.

**Table 3:** **Comparison of time series data among the OD (off-duty), 2NS (two night shifts) and 4NS (four night shifts) groups**

**Variables (mean ± SD)**	**Group (** ***n*** **= 23, 20, 16)**	**Time 1**	**Time 2**	**Time 3**	**Time 4**	**F(2,56)**	***P-*** **value**
Sleep related hormone							
Prolactin (ng/ml)	Off-duty	9.7 ± 3.0	8.5 ± 3.5	8.6 ± 2.9	9.8 ± 3.7	6.31	0.003^a^
	Two night shifts	11.8 ± 6.0	12.5 ± 7.0	13.4 ± 7.0	14.9 ± 6.6		
	Four night shifts	8.1 ± 2.2	8.2 ± 3.0	9.6 ± 2.9	11.1 ± 3.5		
Growth hormone (ng/ml)	Off-duty	1.3 ± 4.0	1.3 ± 2.0	0.5 ± 1.3	1.2 ± 2.6	0.36	0.697
	Two night shifts	1.4 ± 3.0	1.0 ± 1.6	0.6 ± 1.0	1.9 ± 2.4		
	Four night shifts	1.1 ± 0.8	0.9 ± 0.5	0.6 ± 0.9	1.1 ± 1.1		
Thyrotropin (mIU/L)	Off-duty	1.0 ± 0.6	1.0 ± 0.6	1.0 ± 0.6	1.1 ± 0.6	6.64	0.003^b^
	Two night shifts	1.3 ± 0.7	1.5 ± 0.7	1.9 ± 0.9	2.0 ± 0.7		
	Four night shifts	1.0 ± 0.4	1.4 ± 0.5	1.7 ± 0.7	2.1 ± 0.8		
Cortisol (μg/dl)	Off-duty	5.7 ± 3.4	6.0 ± 2.8	4.9 ± 2.8	5.2 ± 2.9	0.12	0.884
	Two night shifts	5.5 ± 2.2	5.9 ± 2.3	4.5 ± 3.0	4.4 ± 2.1		
	Four night shifts	5.4 ± 3.1	5.3 ± 3.0	5.2 ± 2.5	5.1 ± 2.5		

Scheffé’s post-hoc test: ^a^OD group < 2NS group, 4NS group < 2NS group; ^b^OD group < 2NS group, OD group < 4NS group.

**Figure 2:**
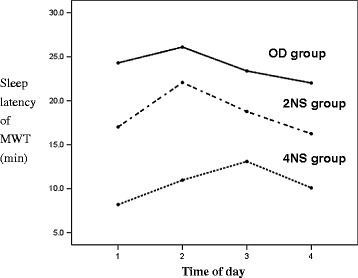
**Mean sleep latency of MWT as a function of time of day.** Mean ± standard deviation of the mean for sleep latency in minutes, for OD, 2NS, and 4NS groups for each time. Between groups comparisons: F (2, 56) = 11.03, *p <* 0.001. Within OD group: F_(3, 66)_ = 2.00, *p* = 0.126; within 2NS group: F_(3, 57)_ = 1.18, *p* = 0.326; within 4NS group: F_(3, 45)_ = 1.17, *p* = 0.331. MWT, Maintenance of Wakefulness Test; 2NS, two consecutive night shifts; 4NS, four consecutive night shifts; OD, off-duty.

**Figure 3:**
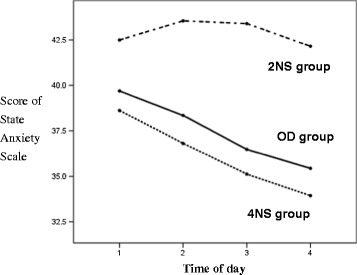
**Mean scores of State Anxiety Scale as a function of time of day.** Mean ± standard deviation of the mean for OD, 2NS, and 4NS groups for each time. Between groups comparisons: F_(2, 56)_ = 5.18, p = 0.009. Within OD group: F_(3, 66)_ = 5.92, *p* = 0.001 (1 > 3, 1 > 4, 2 > 4); within 2NS group: F_(3, 57)_ = 0.61, *p* = 0.610; within 4NS group: F_(3, 45)_ = 7.83, *p* < 0.001 (1 > 3, 1 > 4, 2 > 4). 2NS, two consecutive night shifts; 4NS, four consecutive night shifts; OD, off-duty.

**Figure 4:**
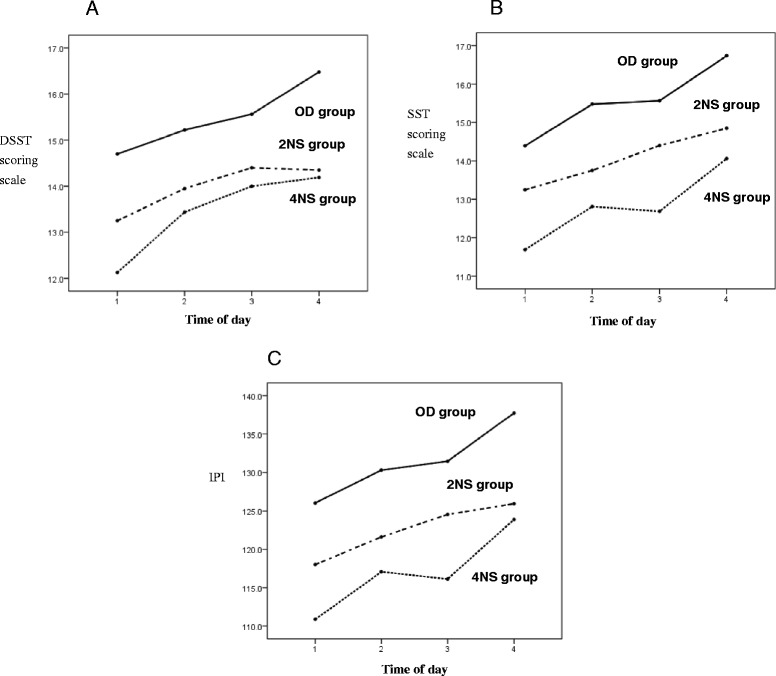
**DSST, SST, and IPI scoring scale as a function of time of day.** Mean ± standard deviation of the mean for OD, 2NS, and 4NS groups for each time. **A)** Between groups comparisons for DSST scoring scale: F_(2,56)_ = 4.72, *p* = 0.013. Within OD group: F_(3, 66)_ = 10.11, *p* < 0.001 (1 < 3, 1 < 4, 2 < 4, 3 < 4); within 2NS group: F_(3, 57)_ = 2.96, *p* = 0.040 (1 < 2, 1 < 3); within 4NS group: F_(3,45)_ = 5.63, *p* = 0.002 (1 < 2, 1 < 3, 1 < 4). **B)** Between groups comparisons for SST scoring scale: F(2, 56) = 6.28, *p* = 0.003. Within OD group: F(3, 66) = 8.85, *p* < 0.001 (1 < 2, 1 < 3, 1 < 4, 2 < 4, 3 < 4); within 2NS group: F(3, 57) = 4.52, *p* = 0.007 (1 < 3, 1 < 4); within 4NS group: F(3, 45) = 4.23, *p* = 0.010 (1 < 2, 1 < 4, 3 < 4). **C)** Between groups comparisons for IPI scoring scale: F(2, 56) = 7.21, *p* = 0.002. Within OD group: F(3, 66) = 15.11, *p* < 0.001 (1 < 2, 1 < 3, 1 < 4, 2 < 4, 3 < 4); within 2NS group: F(3, 57) = 6.26, *p* < 0.001 (1 < 3, 1 < 4); within 4NS group: F(3,45) = 7.25, *p* < 0.001 (1 < 2, 1 < 4, 2 < 4, 3 < 4). DSST, Digit Symbol Substitution Test; SST, Symbol Searching Test; IPI, information process index; 2NS, two consecutive night shifts; 4NS, four consecutive night shifts; OD, off-duty.

**Figure 5:**
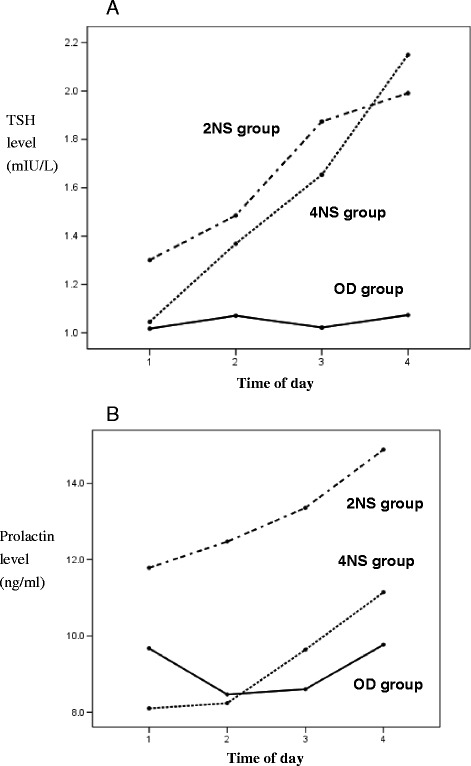
**Mean TSH and prolactin level as a function of time of day.** Mean ± standard deviation of the mean for OD, 2NS, and 4NS groups for each time. **A)** Between groups comparisons for TSH level: F_(2,56)_ = 6.64, *p* = 0.003. Within OD group: F_(3, 66)_ = 0.55, *p* = 0.652); within 2NS group: F_(3, 57)_ = 9.97, *p* < 0.001 (1 < 2,1 < 3,1 < 4, 2 < 3, 2 < 4); within 4NS group: F_(3, 54)_ = 30.77, *p* < 0.001 (1 < 2,1 < 3,1 < 4, 2 < 3, 2 < 4, 3 < 4). **B)** Between groups comparisons for prolactin level: F_(2,56)_ = 6.31, *p* = 0.003. Within OD group: F_(3, 66)_ = 3.33, *p* = 0.025 (1 > 3, 3 < 4); within 2NS group: F_(3, 57)_ = 2.44, *p =* 0.073; within 4NS group: F_(3, 45)_ = 8.68, *p* < 0.001 (1 < 4, 2 < 4, 3 < 4). 2NS, two consecutive night shifts; 4NS, four consecutive night shifts; OD, off-duty; TSH, thyrotropin.

The OD group had a significantly longer MSL of the MWT compared with the night shift groups. The MSL of the MWT in the 2NS group was longer than that in the 4NS group; however, there was no significant time-of-day effect in either group (Figure 2). The 2NS group had higher anxiety scale scores than did the OD and 4NS groups, and this elevated anxiety persisted throughout the day (Figure 3). The OD group performed better on the SST and DSST than did either night shift group. All groups showed a trend toward improved performance on the IPI, SST and DSST as the day progressed, but the learning effect in the OD and 4NSs groups was more pronounced than that in the 2NS group (Figure 4 A–C). Regarding sleep-related hormones, the PRL level in the 2NS group was significantly higher than in the other two groups. In addition, TSH levels were significantly higher in both night shift groups than in the OD group, and remained significantly elevated throughout the day (Figure 5 A, B).

## Discussion

The results suggest that there were no significant differences among the three groups in sleep propensity as measured by the MSLT or in sleepiness as measured by the SSS. The capacity to maintain wakefulness was best in the OD group and worst in the 4NS group. The 2NS group had higher state anxiety scores than did the other two groups, and this elevated state persisted throughout the day. Neuropsychological assessments revealed no significant differences among the groups in WCST performance. Performance of perceptual and motor tasks, as measured by the DSST and SST, was better in the OD group than in either night shift group. Although all groups showed a trend toward improved perceptual and motor abilities during the daytime, the improvements were more significant in the OD group and the 4NS group than in the 2NS group.

Latency in the MSLT reflects the propensity to fall asleep during the daytime. Healthy participants without emotional or sleep disturbances who are allowed to fall asleep under appropriate conditions can often achieve stage one sleep within 10 min according to the MSLT [15]. Although the night shift groups experienced daytime sleep restriction after consecutive night shifts, the increased sleep debt did not affect sleep latency when participants were motivated to go to sleep. Accordingly, there were no statistically significant differences in MSL of the MSLT among the three groups in the study. However, the ability to maintain wakefulness decreased with longer periods of consecutive night work. This may be explained by a phase shift in the circadian rhythms of the nurses, and such an explanation is supported by a gradually decreasing latency in the MWT during the daytime after two to four consecutive night shifts. A significant improvement in cognitive performance in the nighttime on the fourth day after consecutive night shifts has been reported in previous studies [3,16,17]. We did not evaluate sleep quality in the daytime when nurses were working night shifts. Although there was no statistically significant difference in the average self-reported TST among the three groups, it is possible that poor sleep quality in the daytime when working consecutive night shifts caused a sleep debt, which then affected the ability to maintain wakefulness during the daytime of the off day after consecutive night shifts.

We found no differences in sleepiness among the three groups, although the nurses who worked more, rather than fewer, consecutive night shifts had a significantly decreased capacity to maintain wakefulness, as measured by the MWT during the daytime. Performance on the VAT in the night shift groups was also worse than in the OD group. Therefore, after consecutive night shifts, the nurses’ performance on attentive tasks decreased significantly, and their daytime levels of alertness were also lower, even though there was no difference in subjective sleepiness among the groups. This finding is compatible with previous studies [18,19] that suggest people frequently underestimate the impact of sleep restriction on cognitive function. In addition, night shift work has been linked to chronic partial sleep deprivation [20], and chronically deprived persons frequently have the subjective impression that they have adapted to this situation because they do not feel particularly sleepy [21].

The decrements to cognitive performance that result from sleep restriction in healthy adults are consistent with the effects of sleep restriction on physiological sleep propensity measures (MWT, MSLT) [21,22]. It is reasonable, therefore, to presume that cognitive performance in the 2NS group following sleep restriction in the daytime would be better than that in the 4NS group. However, the results of performance on perceptual and motor abilities in the two groups were not statistically different. Although a time-of-day effect on improvements in performance of perceptual motor coordination was also observed in the 2NS group, this effect was not as prominent as in the other two groups.

The 2NS group experienced acute changes in their wake-sleep cycles during the night shifts. Some studies [3,16,17] have indicated adaptation to night shifts on the fourth day, as demonstrated by improvements in cognitive performance at night. Accordingly, destabilization of the homeostatic-circadian two-process model of sleep regulation would be expected to be more pronounced in the 2NS group than in the 4NS group during the daytime after consecutive night shifts. Sleep deprivation studies [2] have shown diverse impacts on cognitive performance as well as mood due to destabilization of the waking state. This may explain why the 2NS group had higher state anxiety scale scores than did the 4NS group and showed persistent elevation of state anxiety throughout the day. Two consecutive night shifts following sleep restriction may be more emotionally stressful than four, and may result in a state of hyperarousal that could contribute to decreases in both performance of attentional tasks and the learning effect [18,23].

The WCST can be considered a measure of “executive function” [24]. In this study, there was no difference in the ability to perform the WCST among the three groups. That is, the ability to shift cognitive strategies in response to changing environmental contingencies was not affected during the day of sleep restriction.

As for sleep-related hormones, the levels of TSH in the night shift groups were higher than those in the OD group, and remained elevated throughout the daytime. The level of PRL in the 2NS group was higher than that in the other two groups, but there were no significant changes in GH and cortisol levels during the day among the three groups.

Cortisol release is mainly controlled by the circadian rhythm, which peaks in the early morning and declines throughout the daytime to a nadir in the late evening [8]. It has been reported that experiencing sleep deprivation the previous day does not affect circadian rhythm-related cortisol release [25]. TSH is regulated by both sleep and the circadian rhythm, and the level is low during the daytime and gradually increases toward evening, reaching a maximum just prior to the onset of sleep [4]. Sleep deprivation causes an elevation of nocturnal TSH, and elevated TSH levels persist into the daytime because of the prolonged half-life of this hormone [26].

In this study, the participants in the night shift groups experienced sleep deprivation before the test day, which should not have affected circadian rhythm-related cortisol release. However, some studies have reported an elevation in evening cortisol levels compared with controls either under chronic sleep deprivation [27,28] or acute sleep loss [7]. In addition, stress has been associated with activation of the hypothalamic-pituitary-adrenal axis, and corticotropin- releasing hormone and cortisol, products of the hypothalamus and the adrenal glands, respectively, are known to cause arousal and sleeplessness [29]. It is possible that the 2NS group had a higher cortisol level than did the other two groups, which would be consistent with the group’s higher state anxiety scores. The negative findings may be due to the small sample size, and it would be interesting to study further the impact of consecutive night shifts on mood and cortisol levels during the day following sleep restriction.

The TSH levels in the night shift groups were higher than those in the OD group and were also elevated throughout the day. There was no difference in TSH levels between the night shift groups, which indicates that daytime levels of TSH may be unaffected by the number of consecutive night shifts worked. GH and PRL secretion are only triggered by sleep onset [8]; therefore, there was no significant change in GH during the daytime among the three groups. However, the PRL levels in the 2NS group were higher than those in the 4NS group. The result suggests that rotating night shifts too quickly may have an impact on PRL levels during the following day’s sleep restriction, and this would also be an interesting topic for future research.

There are some limitations to this study. First, because the tasks were performed in an experimental setting, the results of the neuropsychological findings cannot be generalized to real-life practice. Second, we excluded nurses who used hypnotics or regularly drank coffee, i.e., those who may be least tolerant of shift work. These exclusions may have underestimated the daytime results. Third, we did not collect data on when the nurses slept while working nights, which may have affected how sleepy they were during the 8-h daytime test period. Fourth, the sample size in each group was small. Fifth, we used venipuncture instead of indwelling catheters when collecting blood samples. This may have resulted in discomfort that could lead to overestimation of cortisol levels. Nor did we record the stage of participants’ menstrual cycles, which may have led to an under- or overestimate of PRL levels in the follicular/ovulatory and luteal phases, respectively [30]. Sixth, not all participants had the same shift work schedule before entering the study; this may also have confounded the results. Seventh, blood samples were not collected in the early morning when cortisol release should be reaching peak level. This could have masked differences in cortisol production among the study groups. This was because our study was designed to evaluate physiological changes during the daytime after working consecutive night shifts and to avoid cortisol concentrations’ being influenced by an acute effect. Finally, we did not evaluate chronotype, depression, or psychosocial work characteristics, which may have confounded the results.

## Conclusions

The nurses who worked two, versus four, consecutive night shifts had higher emotional stress during the day after those shifts. This stress may, in turn, have led to a decrease both in performance on attentional tasks and in learning effect, and possibly an impact on PRL level. We suggest that rotating night shifts too quickly may cause anxiety and decreased attentional performance, and may impact PRL levels during the day after night shifts. It is possible that the 2NS group had a higher cortisol level than did the other two groups, which would be consistent with the group’s higher state anxiety scores. The negative findings may be due to the small sample size. It would be interesting to study further the impact of consecutive night shifts on mood and cortisol level during the daytime after sleep restriction.

## Abbreviations

DSST: Digit symbol substitution test
GH: Growth hormone
IPI: Information process index
MSL: Mean sleep latency
MSLT: Multiple sleep latency test
MWT: Maintenance of wakefulness test
2NS: Two consecutive night shifts
4NS: Four consecutive night shifts
OD: Off-duty
PRL: Prolactin
SSS: Stanford sleepiness scale
SST: Symbol searching test
STAI: State-trait anxiety inventory
TSH: Thyrotropin
TST: Total sleep time
WCST: Wisconsin card sorting test
VAT: Visual attention tasks
